# Stereotactic Body Radiotherapy for Low-Risk Prostate Cancer: A Ten-Year Analysis

**DOI:** 10.7759/cureus.1668

**Published:** 2017-09-09

**Authors:** Alan Katz

**Affiliations:** 1 Flushing radiation

**Keywords:** stereotactic radiotherapy, prostate, carcinoma

## Abstract

Objective

This study represents the first 10 year analysis of the efficacy and toxicity of stereotactic body radiotherapy (SBRT) in the treatment of early low-risk prostate cancer.

Materials and methods

Two hundred and thirty males were treated with Cyberknife SBRT to a dose of 35 Gray (Gy) or 36.25 Gy in five consecutive days. All patients had a Gleason score of six and a PSA < 10ng/ml. Median follow-up is nine years. The median age was 69.5 years and median prostate specific antigen (PSA) was 5.6ng/ml. The treatment was delivered with homogeneous planning with a dose prescription of 82-87% of the maximum dose to cover the planning target volume (PTV).

Results

Ten-year biochemical disease free survival was 93% with either dose. Local control was 98.4%. Median prostate specific antigen (PSA) dropped to 0.1 by five years and has remained there. Toxicity was mild with 10% of patients having Grade two-three late urinary toxicity and 4% having the late grade two rectal toxicity. Mean Expanded Prostate Cancer Index Composite (EPIC) Quality of Life (QOL) scores declined initially for bowel and urinary domains, but recovered to baseline, where they remain. EPIC sexual scores have declined by 40%.

Discussion/Conclusions

Stereotactic body radiotherapy to a dose of 35 Gy-36.25 Gy is an effective treatment for early low-risk prostate cancer, with acceptably low toxicity. There appears to be no benefit to increasing the dose beyond 35 Gy. Ten-year biochemical disease free survival appears to be higher than with standard intensity modulated radiotherapy (IMRT).

## Introduction

Over the last 10 years, many reports of stereotactic body radiotherapy (SBRT) treatments for early prostate cancer, in which high fractional doses are delivered in four or five fractions, have appeared in the literature [1-7]. The effects of doses of 35 Gy to 50 Gy in five fractions have been assessed. With follow-up up to seven years, these studies, mostly involving low- and intermediate- risk patients have shown excellent biochemical control with low rates of toxicity. The sensitivity of the prostate cancer to dose per fraction probably accounts for these favorable results [8]. Although the outcomes to date have been favorable, a legitimate concern is that the reported follow-up is not long enough to identify all the benefits and pitfalls of this treatment. In this study, we address these concerns by reporting a large cohort of low-risk patients with up to 10-years of follow-up.

## Materials and methods

From 2006-2008, we treated 230 low-risk prostate cancer patients with CyberKnife SBRT. The patients received a dose of 3500 to 3625 cGy in five daily fractions. All patients had a Gleason score of six. The median prostate specific antigen (PSA) was 5.6 ng/ml. The median age was 69.5 years (range 47-86 years). Ninety percent of the patients were T1c and 10% were T2a. The patients had one to nine positive cores on 12-core biopsy. The median prostate volume was 45 cc (range 18-103 cc). All patients were planned with a computerized tomography (CAT) scan fused with a magnetic resonance imaging (MRI) scan. The first 42 patients received a dose of 35 Gy in five daily fractions to the PTV and the rest received 36.25 Gy, also in five daily fractions. The treatment dose was defined to cover 95% of the PTV. The PTV was defined as a 5-m expansion of the actual prostate volume, reduced to 3 mm toward the rectum. Seminal vesicles were included in the treatment volume for lesions at the base. The dose prescribed was 82-87% of the maximum dose in the prostate. The mean dextrose (D50) to the rectum and bladder was 43% of the maximum dose. All patients received 500 mg of Amifostine solution instilled into the rectum 15 minutes before the treatment.

The median follow-up for the entire cohort was 108 months (zero–120 months). In general, PSA values were obtained at baseline, and prospectively at three-month post-treatment intervals during the first two years and at six-months intervals thereafter. The PSA relapse definition used was the current adopted standard of care Phoenix definition (i.e., nadir + 2) [9]. Biochemical disease-free survival (BDFS) was calculated with the Kaplan–Meier method. A benign PSA bounce was recorded when PSA rose by > 0.2 ng/mL above the post-treatment nadir and subsequently returned to nadir levels or below.

Toxicity

Acute and late genitourinary (GU) and gastrointestinal (GI) toxicity were scored according to the criteria set by the Radiation Therapy Oncology Group (RTOG) [10].

Quality of life

Prior to the treatment, all the patients completed the initial Expanded Prostate Cancer Index Composite (EPIC) questionnaire to evaluate urinary, bowel, and sexual quality of life (QOL) [11]. At each subsequent time, the patients were requested to fill out the EPIC questionnaire to assess follow-up QOL.

Statistical analysis

The primary endpoint of the study was interval to BDFS. Kaplan–Meier survival method was used to estimate BDFS and log-rank p-values were used to compare the distributions. One tailed test was used to determine p value for the difference in toxicity.

## Results

**Figure 1:**
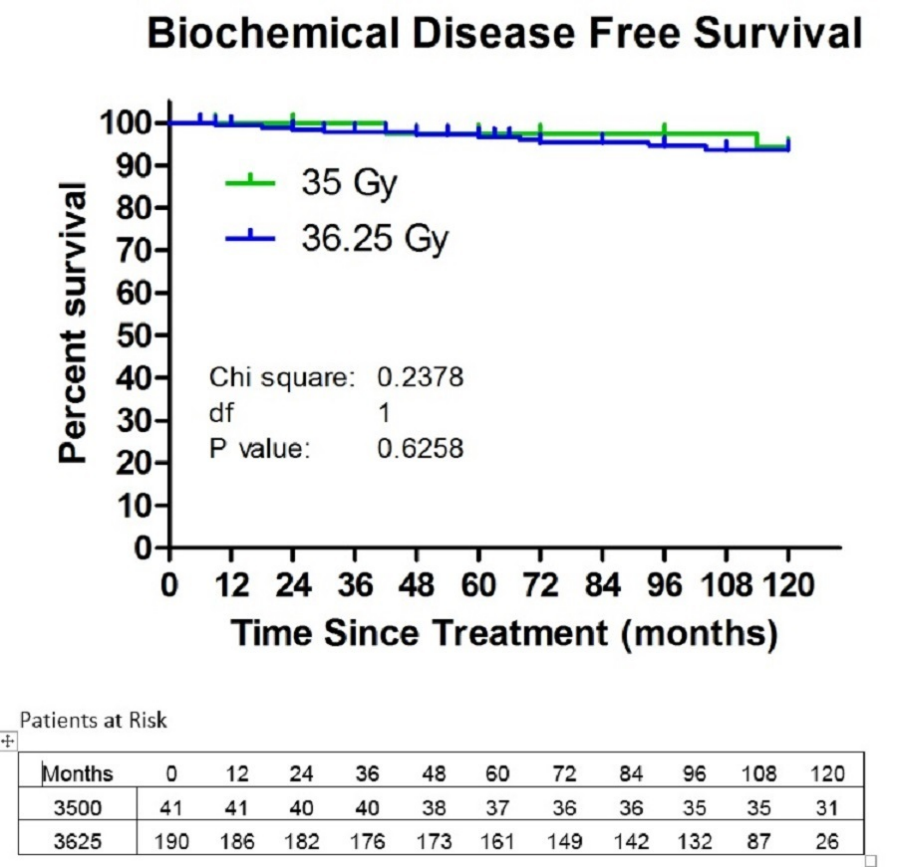
Biochemical disease-free survival (BDFS) in the patients treated with 35 or 36.25 Gy. The 10-year Kaplan-Meier estimate of BDFS was 94.4% for the patients treated with 35 Gy and 93.4% for the patients treated with 36.25 Gy.

**Figure 2:**
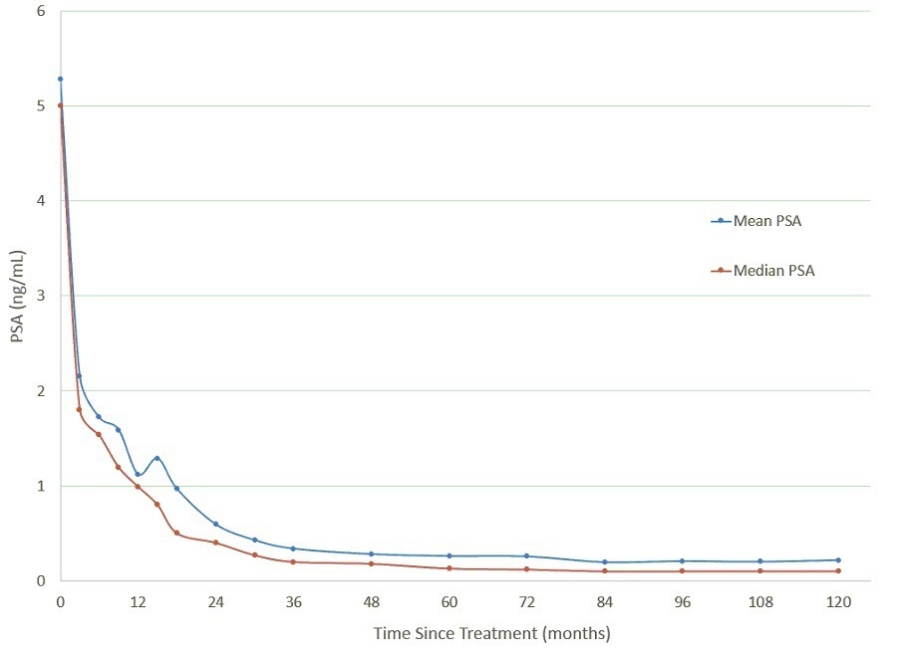
Mean (blue) and median (red) prostate specific antigen (PSA) value as a function for months since treatment.

The 10-year actuarial biochemical disease free survival was 93.7% (Figure 1). Local control was 98.4%. The median PSA level dropped to 0.1 ng/ml at 48 months. At 10-years, the median PSA remains at 0.1 ng/ml (Figure 2). There was no difference in control or median PSA between the two doses of 35 Gy and 36.25 Gy. Twenty-one percent of the patients experienced a PSA bounce up to the first six years. None of the patients died of prostate cancer.

The toxicity was mild. Grade 1-2 acute urinary toxicity was noted in 78% of the patients; 59% had acute Grade 1-2 rectal toxicity. There was no Grade 3-4 toxicity. Late urinary toxicity occurred in 9% (Grade 2) and 3% (Grade 3) of the patients. Late urinary toxicity Grade 2-3 appeared higher in the high-dose group (4% at 35 Gy vs 15% at 36.25 Gy), and this difference approached significance (p=0.07). Four percent of the patients had Grade 2 rectal toxicity with no Grade 3-4 events. There was no clear difference in late rectal toxicity rates between the two doses. For patients who were potent prior to SBRT, 56% remained potent at last follow-up.

**Figure 3:**
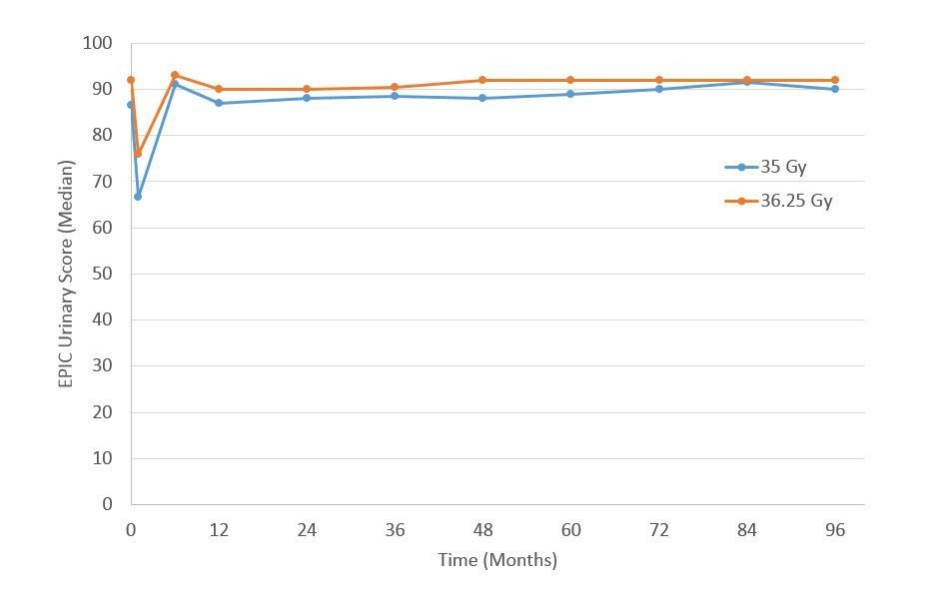
Expanded Prostate Cancer Index Composite (EPIC) urinary quality of life as a function of time since the treatment for the 35 and 36.25 Gy cohorts.

**Figure 4:**
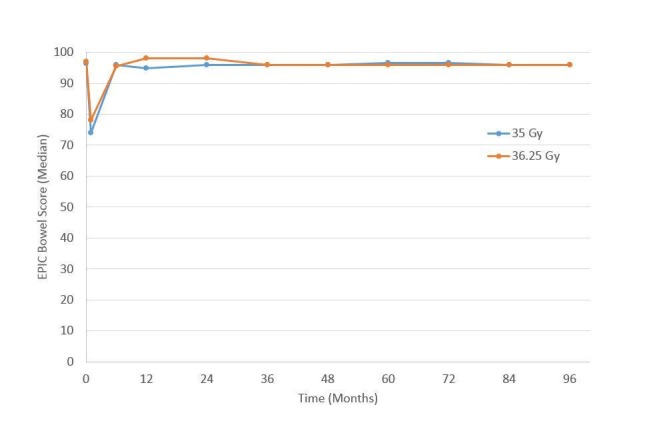
Expanded Prostate Cancer Index Composite (EPIC) bowel quality of life as a function of time since the treatment of 35 and 36.25 Gy cohorts.

**Figure 5:**
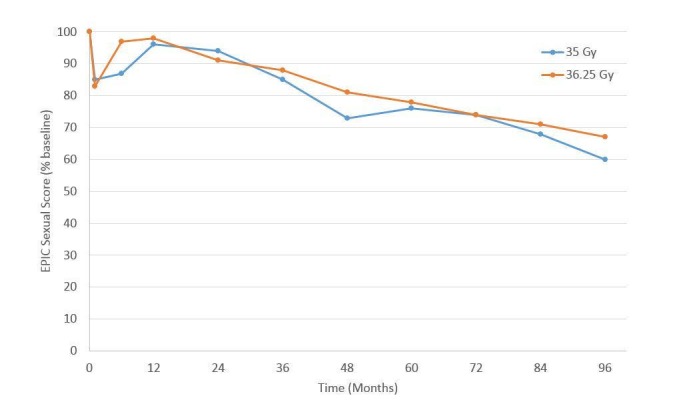
Expanded Prostate Cancer Index Composite (EPIC) sexual quality of life for the patients with good pre-treatment function, as a function of time since the treatment of 35 and 36.25 Gy cohorts.

For urinary (Figure 3) and bowel (Figure 4) EPIC domains, the mean EPIC scores decreased acutely and then gradually rose back to baseline by one-year. After one-year, mean EPIC scores extending, out to eight years did not differ significantly from baseline. The EPIC sexual QOL declined by 23% at six–12 months and decreased by 38% by eight years (Figure 5). There was no significant difference in EPIC bowel, sexual, or urinary scores between 35 Gy or 36.25 Gy at any point.

## Discussion

At ten-years, this report demonstrates excellent long-term control of low-risk prostate cancer with low toxicity. These outcomes compare quite favorably to the patients treated with IMRT to 81 Gy. The two reports of standard IMRT with this long-term follow-up show biochemical control rates of 81-85% of low-risk patients [12-13]. A ten-year study of such patients with high dose rate (HDR) brachytherapy demonstrated a 92% control rate, quite similar to our patients [14]. This supports the notion that a dose of 81 Gy in 45 fractions, or 178 biologically effective dose (BED), may not be enough to maximize tumor control. Since HDR and SBRT seem to yield higher control rates, it is highly likely that the equivalent dose delivered with these two modalities is significantly higher than 81 Gy. This is probably a function of the low alpha-beta ratio of prostate cancer. In fact, an alpha-beta ratio of 1.5 Gy would mean that 35-36.25 Gy in five fractions yields an equivalent dose of 90-95 Gy at 1.8 Gy per fraction, or 200-212 Gy BED.

If a dose escalation with SBRT delivers higher control rates, the next question is at what dose will the maximum benefit occur. Based on our data, 35 Gy in five fractions achieves the maximum benefit as our higher dose showed no further benefit. Using an alpha-beta ratio of 1.5 Gy, our 35-Gy dose has a BED of 200 Gy. This is consistent with a recent meta-analysis of prostate cancer patients treated with various radiation modalities, which showed a maximum benefit for 200 BED, with only more toxicity for higher doses [15].

Consistent with the above, our data also show higher urinary toxicity with 36.25 Gy, which has a BED of 212 Gy. This strongly suggests that 35 Gy may be the optimal dose to employ in lower risk patients and that higher doses being employed today will only yield more toxicity with no further benefit. Whether higher doses may benefit higher risk patients remains an open question. These results will need to be confirmed by the maturation of other trials and possibly randomized trials between SBRT and other radiation modalities.

## Conclusions

Stereotactic body radiotherapy (SBRT) of 35-36.25 Gy in five fractions yields excellent control with low toxicity in low-risk prostate cancer patients. Further randomized trials are necessary to compare its efficacy with other forms of radio therapy (RT) and to determine the optimal SBRT dose. This study suggests that SBRT is superior to standard IMRT in terms of control with no increase in late toxicity.
